# Skeletal development in blue‐breasted quail embryos

**DOI:** 10.1111/asj.13159

**Published:** 2019-01-17

**Authors:** Yoshiaki Nakamura, Yoshifumi Nakane, Masaoki Tsudzuki

**Affiliations:** ^1^ Laboratory of Animal Breeding and Genetics Graduate School of Biosphere Science Hiroshima University Higashi‐Hiroshima Hiroshima Japan; ^2^ Japanese Avian Bioresource Project Research Center Hiroshima University Higashi‐Hiroshima Hiroshima Japan; ^3^ Institute of Laboratory Animals Graduate School of Medicine Kyoto University Sakyou‐ku Kyoto Japan

**Keywords:** blue‐breasted quail, embryos, skeletal development, whole‐mount skeletal staining

## Abstract

The blue‐breasted quail (*Coturnix chinensis*), the smallest species of quail with short generation interval and excellent reproductive performance, is a potential avian research model. A normal series of skeletal development of avian embryos could be served as a reference standard in the fields of developmental biology and teratological testing as well as in the investigation of mutation with skeletal abnormalities and in the study of the molecular mechanisms of skeletal development through genome manipulation. Furthermore, ossification sequence shows a species‐specific pattern and has potential utility in phylogeny. However, data on the skeletal development of blue‐breasted quail embryos are scarce. Here, we established a series of normal stages for the skeletal development of blue‐breasted quail embryos. Cartilage and ossified bones of blue‐breasted quail embryos were stained blue and red with Alcian blue 8GX and Alizarin red S, respectively. The time and order of chondrification and calcification of their skeletons were documented every 24 hr from 3 to 17 days of incubation, and a 15‐stage series of skeletal development was created. Moreover, a comparative study with the Japanese quail (*Coturnix japonica*) demonstrated that ossification sequence differed significantly between these two species.

## 1 INTRODUCTION

The blue‐breasted quail (*Coturnix chinensis*) is a species of old‐world quail in the family of Phasianidae, ranging in the wild from India, Southeast Asia, and the south‐eastern area of China (Uchida & Shimasaki, 1987; Yamashina, 1986). This species is sometimes kept as a pet because of its small body size and variable plumage colors. The blue‐breasted quail is the smallest in the quail species (~50 g). Its body size is about half that of the Japanese quail (*Coturnix japonica*), and it is relatively easy to handle. The egg production rate of the blue‐breasted quail is over 70% (Tsudzuki, 1994). A primary advantage of using the blue‐breasted quail as an avian research model is its compact size. In fact, three times as many of these birds can fit in an animal room as the much larger Japanese quail (Tsudzuki, 1994). Thus, the use of the blue‐breasted quail as an avian model allows researchers to reduce the cost, space, and labor for breeding. Because of these advantages, the blue‐breasted quail has been used in the various research fields, for example, developmental biology, genetics, reproduction, behavior, and immunology for more than 20 years (Adkins‐Regan, 2016; Araguas, Sanz, Viñas, & Vidal, 2018; Kageyama, Takenouchi, Kinoshita, Nakamura, & Tsudzuki, 2018; Ma et al., 2012; Nishibori, Tsudzuki, Hayashi, Yamamoto, & Yasue, 2002; Ono et al., 2005; Parker et al., 2017; Tsudzuki, 1995a, 1995b; Wells, Parker, Kiess, & McDaniel, 2012).

The skeletal system that is the system of bones, associated cartilages and joints serves as a framework for the body. It is well known that the skeletal system performs vital functions for terrestrial vertebrates, for example, support of the body, internal organ protection, locomotion, blood cell production, and minerals and fat storage and release. In birds, skeletal system has evolved over time to increase the ability to fly. Avian skeletal system is extremely lightweight due to their hollow bones but tough enough to withstand the stresses of flying. One major morphological feature of the avian skeletal system is that some parts of vertebrae are fused into a single ossification. Hence, birds usually have a smaller number of bones than mammals or reptiles. Most of avian species have the keeled sternum that provides a large surface area for the strong attachment of the breast muscles used in flying or swimming. Birds lack teeth and the heavy jaws to support them, and instead have beaks, which is much more lightweight.

There are two essential processes of skeletogenous during embryonic development, that is, intramembranous ossification and endochondral ossification. Intramembranous ossification produces many of the craniofacial bones directly from mesenchymal tissues. On the other hands, endochondral ossification is the principle embryonic process of bone formation in which long bones develop by replacing cartilage templates. The replacement from cartilage to bone is tightly coupled with chondrocyte, osteoblast, and vascular differentiation. In birds, ossification spans the latter two‐thirds of embryonic development and continued after hatching.

Application of avian embryos in teratological tests has been proposed as one of

the alternative methods (Hashizume et al., 1992). Thalidomide was widely prescribed to pregnant women as a sedative but was found to be teratogenic, causing multiple birth defects, for example, malformation of limbs and defects of ears, eyes, heart, kidney, and other internal organs (Knobloch & Rüther, 2008). Chicken embryos have been used as a well‐established model system for studying the molecular mechanisms of thalidomide teratogenicity (Debock & Peters, 1963; Knobloch, Shaughnessy, & Rüther, 2007; Therapontos, Erskine, Gardner, Figg, & Vargesson, 2009). Thalidomide initiates its teratogenic effects by binding to cereblon and inhibiting the associated ubiquitin ligase activity in limbs of chicken embryos (Ito et al., 2010). The list of the steps comprising normal skeletal development of blue‐breasted quail embryos is required when this species is used to teratogenic tests. Moreover, skeletogenous list of the blue‐breasted quail would be useful in identification of mutations with skeletal defects and for manipulation of their genome for studying molecular mechanisms of skeletal development in future.

The purpose of this study is to document the normal staging table of skeletal development in blue‐breasted quail embryos. Thereafter, ossification sequence of blue‐breasted quail embryos was compared with that of Japanese quail embryos.

## 2 MATERIALS AND METHODS

### 2.1 Birds and eggs

Fertilized eggs of wild‐type blue‐breasted quail (Tsudzuki, 1994) were collected within 6 hr after laying and stored at 15°C for ~5 days. They were then incubated at 37.7 ± 0.2°C and a relative humidity of 70% while being tilted at a 90° angle once per hour (MIC‐14C; M's Factory, Nagoya, Japan). Embryos were collected every 24 hr from 3 to 17 days of the incubation. Newly hatched chicks were sacrificed by CO_2_ inhalation. Embryos were staged according to developmental series reported by Nakamura, Nakane, and Tsudzuki (NNT stages; in press). This study was approved by Hiroshima University Animal Research Committee (Permission number: C18‐26) and carried out according to the Guidelines for Proper Conduct of Animal Experiments (Science Council of Japan 2006), the Fundamental Guidelines for Proper Conduct of Animal Experiment and Related Activities in Academic Research Institutions under the jurisdiction of the Ministry of Education, Culture, Sports, Science and Technology (Ministry of Education, Culture, Sports, Science and Technology 2006), and Hiroshima University Animal Experimentation regulations.

### 2.2 Double staining of the embryonic skeleton

Blue‐breasted quail embryo skeletons were double‐stained with Alcian Blue 8GX S (Wako Pure Chemical Industries Ltd., Osaka, Japan) and Alizarin Red S (Wako Pure Chemical Industries Ltd.) for cartilage and ossified bones, respectively. Staining was carried out according to Nakane and Tsudzuki (1999), with minor modifications as described below.

For 3‐ to 6‐day embryos (NNT stages 19–25), they were fixed and stained for 18 hr in a 4:1 mixture of 95% v/v ethanol and acetic acid containing 0.015% Alcian Blue 8GX. Fixed samples were dehydrated three times in 95% v/v ethanol for 3 days then macerated for 3 hr in 1% w/v KOH. After clearing in a glycerol/H_2_O concentration series (25%, 50%, and 75%) for 7 days at each concentration, stained samples were stored in 100% glycerol.

For 7‐ and 8‐day embryos (NNT stages 27 and 29), they were fixed, stained, and dehydrated according to the same procedure as written for 3‐ to 6‐day embryos. After staining of the cartilage for 24 hr, skin and viscera were removed. Samples were stained and macerated for 6 hr in 0.002% Alizarin Red S/1% KOH. Finally, they were cleared and stored in the same procedure described for 3‐ to 6‐day embryos.

For 9‐ to 11‐day embryos (NNT stages 31–33), all procedures are the same as described for 7‐ and 8‐day embryos except for staining period. Ossified bones were stained for 24 hr.

For 12‐ to 16‐day embryos and newly hatched chicks (NNT stages 34–39), skin, and viscera were removed. Subsequent fixation, staining, dehydration, clearing, and storing were performed according to the same procedure as written for 7‐ and 8‐day embryos. Ossified bones were stained for 2 days.

### 2.3 Photography and measurement

Chondrification was visualized with a blue color from Alcian Blue 8GX S staining. Calcification was visualized with a red color from Alizarin Red S staining. They were observed under a stereomicroscope (S9i, Leica Microsystems, Tokyo, Japan). Calcific region was identified as the region where stained with only red in color as described elsewhere (Nakane & Tsudzuki, 1999). On and after 7 days of incubation, the lengths of calcific region as well as full lengths of the humerus, ulna, femur, and tibia were measured using a micrometer as described (Nakane & Tsudzuki, 1999). The percentages of calcific region (length of the red‐stained region/full length of the humerus, ulna, femur, and cervical vertebrae; means ± *SD*) were also calculated as described (Nakane & Tsudzuki, 1999).

## 3 RESULTS

**Figure 1:**
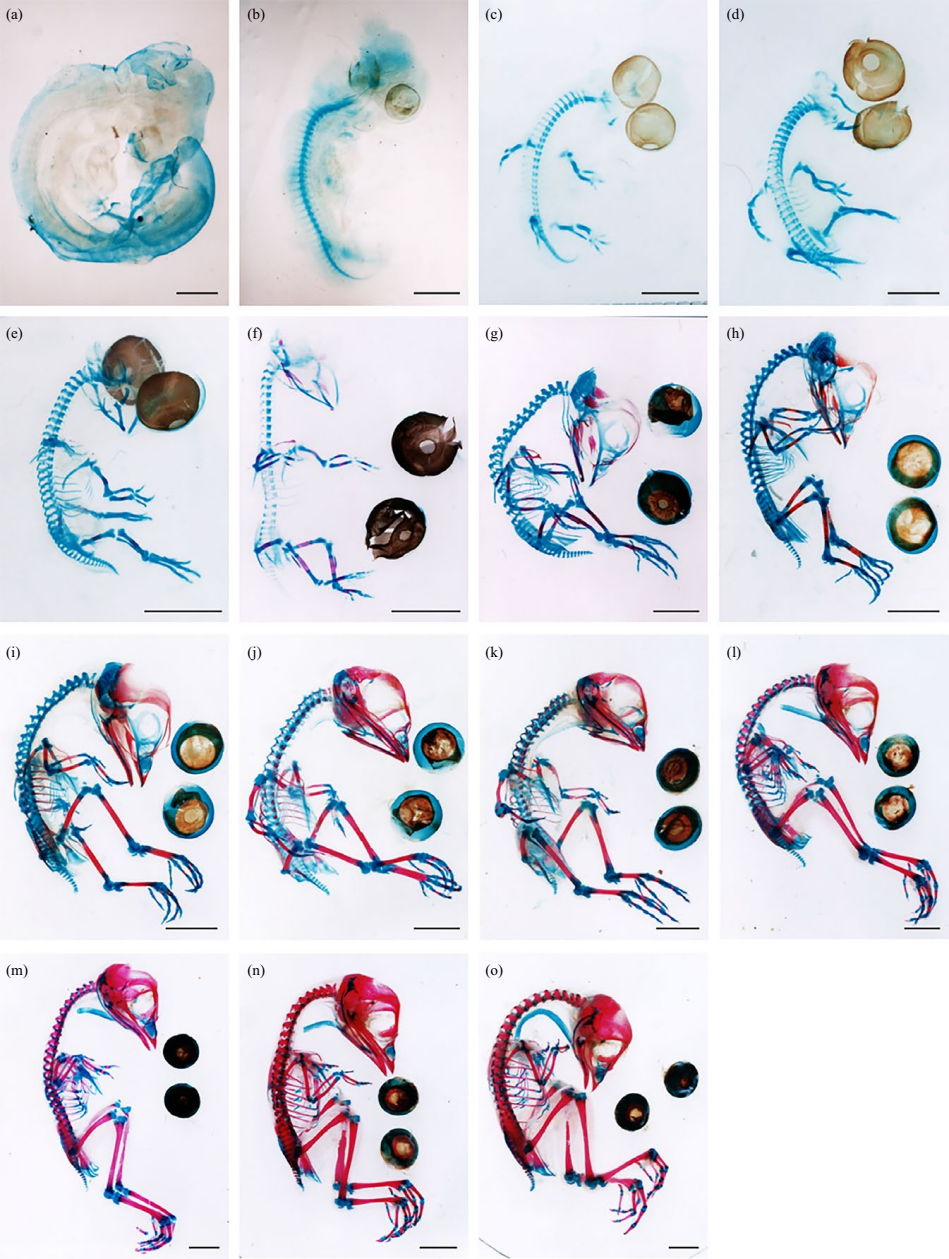
Skeletogenesis of blue‐breasted quail embryos at 3–16 days of incubation and newly hatched chicks. Detailed explanations are provided in the text. (a–n) Embryos prepared daily from 3 to 16 days of incubation. (o) A newly hatched chick. Bar, 1 mm (a); 2 mm (b); 3 mm (c, d); 5 mm (f‐o)

**Figure 2:**
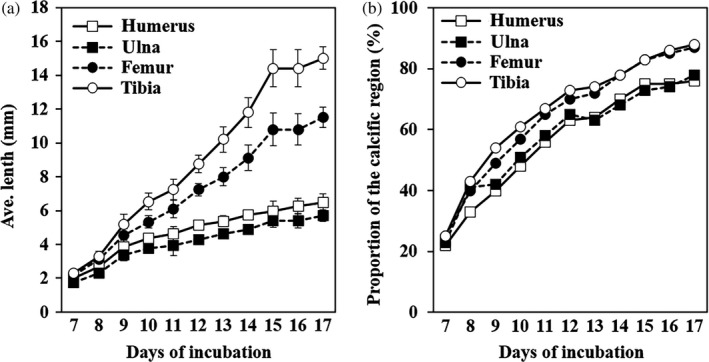
Changes in the average lengths of the humerus, ulna, femur, and tibia, and the percentage of calcific regions in these bones. (a) Average length (*M* ± *SD*) of the humerus, ulna, femur, and tibia. (b) Percentage of calcific regions in the humerus, ulna, femur, and tibia

The sequential development of blue‐breasted quail embryo skeletons is shown in Figure 1. The skeleton was divided into the skull, vertebrae, ribs and sternum, and fore‐ and hindlimbs. The transition from chondrification to ossification in each part of the skeleton is summarized in Tables 1, 2, 3, 4. The average lengths of the humerus, ulna, femur, and tibia are shown in Figure 2a. The percentages of calcification in these bones are shown in Figure 2b. The morphological features of the skeletal development in blue‐breasted quail embryos (3–16 days of incubation) and newly hatched chicks are described below.

**Table 1:** Transition from chondrification to ossification of the skull

Skull bones	Days of incubation														
	3	4	5	6	7	8	9	10	11	12	13	14	15	16	17^a^
Occipital															
Basioccipital	―	〇	〇	〇	〇	〇	●	●	●	●	●	●	●	●	●
Exoccipital	―	〇	〇	〇	〇	〇	●	●	●	●	●	●	●	●	●
Supraoccipital	―	―	―	―	―	―	〇	〇	●	●	●	●	●	●	●
Sphenoid															
Parasphenoid	―	―	―	〇	〇	〇	〇	〇	●	●	●	●	●	●	●
Basisphenoid	―	〇	〇	〇	〇	〇	〇	〇	●	●	●	●	●	●	●
Orbitosphenoid	―	―	〇	〇	〇	〇	〇	〇	●	●	●	●	●	●	●
Temporal															
Squamosal	―	―	―	―	―	●	●	●	●	●	●	●	●	●	●
Petrosal	―	―	〇	〇	〇	〇	〇	〇	●	●	●	●	●	●	●
Parietal	―	―	―	―	―	―	―	●	●	●	●	●	●	●	●
Frontal	―	―	―	―	―	●	●	●	●	●	●	●	●	●	●
Prefrontal	―	―	―	―	―	●	●	●	●	●	●	●	●	●	●
Mesethmoid	―	―	―	―	〇	〇	〇	〇	〇	〇	〇	●	●	●	●
Trabeculae	―	―	〇	〇	〇	〇	〇	〇	〇	―	―	―	―	―	―
Nasal	―	―	―	―	―	●	●	●	●	●	●	●	●	●	●
Premaxillary	―	―	―	―	―	●	●	●	●	●	●	●	●	●	●
Maxillary	―	―	―	―	―	●	●	●	●	●	●	●	●	●	●
Palatine	―	―	―	―	―	―	●	●	●	●	●	●	●	●	●
Vomer	―	―	―	―	―	―	―	―	●	●	●	●	●	●	●
Pterygoid	―	―	―	―	―	●	●	●	●	●	●	●	●	●	●
Jugal	―	―	―	―	―	●	●	●	●	●	●	●	●	●	●
Quadratojugal	―	―	―	―	●	●	●	●	●	●	●	●	●	●	●
Quadrate	―	―	〇	〇	〇	〇	●	●	●	●	●	●	●	●	●
Mandible															
Mandibular	―	―	〇	〇	〇	〇	〇	〇	〇	〇	〇	〇	〇	〇	〇
Dentary	―	―	―	―	―	―	●	●	●	●	●	●	●	●	●
Supra‐angular	―	―	―	―	●	●	●	●	●	●	●	●	●	●	●
Angular	―	―	―	―	●	●	●	●	●	●	●	●	●	●	●
Splenial	―	―	―	―	―	●	●	●	●	●	●	●	●	●	●
Prearticular	―	―	―	―	―	―	●	●	●	●	●	●	●	●	●
Articular	―	―	―	〇	〇	〇	〇	〇	〇	〇	〇	〇	●	●	●
Hyoid apparatus															
Entoglossal	―	―	―	―	―	〇	〇	〇	〇	〇	〇	〇	〇	〇	〇
Basihyal	―	―	〇	〇	〇	〇	〇	〇	〇	〇	〇	〇	〇	〇	〇
Urohyal	―	―	〇	〇	〇	〇	〇	〇	〇	〇	〇	〇	〇	〇	〇
Ceratobranchial	―	―	〇	〇	〇	●	●	●	●	●	●	●	●	●	●
Epibranchial	―	―	〇	〇	〇	〇	〇	〇	〇	〇	〇	〇	〇	〇	〇

―, Stained with neither Alcian Blue 8GX nor Alizarin Red S; 〇, stained blue with Alcian Blue 8GX; ●, partially stained red with Alizarin Red S.

a Newly hatched chicks.

**Table 2:** Transition from chondrification to ossification of the vertebrae

Vertebral bones	Days of incubation														
	3	4	5	6	7	8	9	10	11	12	13	14	15	16	17^a^
Cervical vertebrae															
Upper region															
Centrum	〇	〇	〇	〇	〇	〇	〇	〇	●	●	●	●	●	●	●
Vertebral arch	―	〇	〇	〇	〇	〇	〇	〇	〇	●	●	●	●	●	●
Cervical rib	―	―	―	―	―	〇	〇	〇	〇	●	●	●	●	●	●
Medial region															
Centrum	〇	〇	〇	〇	〇	〇	〇	〇	〇	●	●	●	●	●	●
Vertebral arch	―	〇	〇	〇	〇	〇	〇	〇	〇	●	●	●	●	●	●
Cervical rib	―	―	―	―	〇	〇	〇	〇	〇	●	●	●	●	●	●
Lower region															
Centrum	〇	〇	〇	〇	〇	〇	〇	〇	〇	●	●	●	●	●	●
Vertebral arch	―	〇	〇	〇	〇	〇	〇	〇	〇	●	●	●	●	●	●
Cervical rib	―	―	―	―	〇	〇	〇	〇	〇	●	●	●	●	●	●
Thoracic vertebrae															
Upper region															
Centrum	〇	〇	〇	〇	〇	〇	〇	〇	●	●	●	●	●	●	●
Vertebral arch	―	〇	〇	〇	〇	〇	〇	〇	〇	●	●	●	●	●	●
Medial region															
Centrum	〇	〇	〇	〇	〇	〇	〇	〇	●	●	●	●	●	●	●
Vertebral arch	―	〇	〇	〇	〇	〇	〇	〇	〇	〇	●	●	●	●	●
Lower region															
Centrum	〇	〇	〇	〇	〇	〇	〇	〇	●	●	●	●	●	●	●
Vertebral arch	―	〇	〇	〇	〇	〇	〇	〇	〇	〇	〇	●	●	●	●
Lumbosacral vertebrae															
Upper region															
Centrum	〇	〇	〇	〇	〇	〇	〇	〇	〇	●	●	●	●	●	●
Vertebral arch	―	〇	〇	〇	〇	〇	〇	〇	〇	〇	〇	●	●	●	●
Medial region															
Centrum	〇	〇	〇	〇	〇	〇	〇	〇	〇	〇	●	●	●	●	●
Vertebral arch	―	〇	〇	〇	〇	〇	〇	〇	〇	〇	〇	〇	●	●	●
Lower region															
Centrum	〇	〇	〇	〇	〇	〇	〇	〇	〇	〇	〇	●	●	●	●
Vertebral arch	―	〇	〇	〇	〇	〇	〇	〇	〇	〇	〇	〇	●	●	●
Transverse process	―	―	―	―	―	〇	〇	〇	〇	〇	〇	●	●	●	●
Coccygeal vertebrae															
Upper region															
Centrum	〇	〇	〇	〇	〇	〇	〇	〇	〇	〇	〇	●	●	●	●
Vertebral arch	―	〇	〇	〇	〇	〇	〇	〇	〇	〇	〇	●	●	●	●
Medial region															
Centrum	―	〇	〇	〇	〇	〇	〇	〇	〇	〇	〇	〇	〇	〇	●
Vertebral arch	―	―	〇	〇	〇	〇	〇	〇	〇	〇	〇	〇	●	●	●
Lower region															
Centrum	―	〇	〇	〇	〇	〇	〇	〇	〇	〇	〇	〇	〇	〇	●
Vertebral arch	―	―	〇	〇	〇	〇	〇	〇	〇	〇	〇	〇	〇	〇	●

―, Stained with neither Alcian Blue 8GX nor Alizarin Red S; 〇, stained blue with Alcian Blue 8GX; ●, partially stained red with Alizarin Red S.

a Newly hatched chicks.

**Table 3:** Transition from chondrification to ossification of the ribs and sternum

Ribs and sternum	Days of incubation														
	3	4	5	6	7	8	9	10	11	12	13	14	15	16	17^a^
Ribs															
First vertebral rib	―	―	〇	〇	〇	〇	〇	●	●	●	●	●	●	●	●
Second vertebral rib	―	―	〇	〇	〇	〇	〇	●	●	●	●	●	●	●	●
Uncinate process	―	―	―	―	―	〇	〇	〇	〇	〇	〇	〇	〇	●	●
Third vertebral rib	―	―	〇	〇	〇	〇	●	●	●	●	●	●	●	●	●
Uncinate process	―	―	―	―	―	〇	〇	〇	〇	〇	〇	〇	〇	●	●
Fourth vertebral rib	―	―	〇	〇	〇	〇	●	●	●	●	●	●	●	●	●
Uncinate process	―	―	―	―	―	〇	〇	〇	〇	〇	〇	〇	〇	●	●
Fifth vertebral rib	―	―	〇	〇	〇	〇	●	●	●	●	●	●	●	●	●
Uncinate process	―	―	―	―	―	〇	〇	〇	〇	〇	〇	〇	〇	●	●
Sixth vertebral rib	―	―	〇	〇	〇	〇	●	●	●	●	●	●	●	●	●
Uncinate process	―	―	―	―	―	―	―	〇	〇	〇	〇	〇	〇	●	●
Seventh vertebral rib	―	―	〇	〇	〇	〇	〇	●	●	●	●	●	●	●	●
Third sternal rib	―	―	―	―	〇	〇	〇	〇	〇	〇	●	●	●	●	●
Fourth sternal rib	―	―	―	―	〇	〇	〇	〇	〇	〇	●	●	●	●	●
Fifth sternal rib	―	―	―	―	〇	〇	〇	〇	〇	〇	●	●	●	●	●
Sixth sternal rib	―	―	―	―	〇	〇	〇	〇	〇	〇	〇	●	●	●	●
Seventh sternal rib	―	―	―	―	―	―	―	―	〇	〇	〇	〇	●	●	●
Sternum															
Body	―	―	―	―	〇	〇	〇	〇	〇	〇	〇	〇	〇	〇	●
Manubrium	―	―	―	―	―	―	―	〇	〇	〇	〇	〇	〇	〇	〇
Crest	―	―	―	―	―	―	―	〇	〇	〇	〇	〇	〇	〇	〇
Laterocranial process	―	―	―	―	〇	〇	〇	〇	〇	〇	〇	〇	●	●	●
Inner laterocaudal process	―	―	―	―	〇	〇	〇	〇	〇	〇	〇	〇	●	●	●
Outer laterocaudal process	―	―	―	―	〇	〇	〇	〇	〇	〇	〇	〇	●	●	●

―, Stained with neither Alcian Blue 8GX or Alizarin Red S; 〇, stained blue with Alcian Blue 8GX; ●, partially stained red with Alizarin Red S.

a Newly hatched chicks.

**Table 4:** Transition from chondrification to ossification of the fore‐ and hindlimbs

Fore‐ and hindlimbs	Days of incubation														
	3	4	5	6	7	8	9	10	11	12	13	14	15	16	17^a^
Forelimb															
Scapula	―	―	〇	〇	〇	●	●	●	●	●	●	●	●	●	●
Coracoid	―	―	〇	〇	〇	〇	●	●	●	●	●	●	●	●	●
Clavicle	―	―	―	―	●	●	●	●	●	●	●	●	●	●	●
Humerus	―	〇	〇	〇	●	●	●	●	●	●	●	●	●	●	●
Radius	―	―	〇	〇	●	●	●	●	●	●	●	●	●	●	●
Ulna	―	―	〇	〇	●	●	●	●	●	●	●	●	●	●	●
First metacarpus	―	―	―	〇	〇	〇	〇	〇	〇	〇	〇	〇	〇	〇	〇
Second metacarpus	―	―	〇	〇	●	●	●	●	●	●	●	●	●	●	●
Third metacarpus	―	―	〇	〇	〇	●	●	●	●	●	●	●	●	●	●
First phalanx of the first digit	―	―	―	―	〇	〇	〇	●	●	●	●	●	●	●	●
Second phalanx of the first digit	―	―	―	―	〇	〇	〇	〇	〇	〇	〇	●	●	●	●
First phalanx of the second digit	―	―	―	〇	〇	〇	〇	●	●	●	●	●	●	●	●
Second phalanx of the second digit	―	―	―	―	〇	〇	〇	●	●	●	●	●	●	●	●
First phalanx of the third digit	―	―	―	〇	〇	〇	〇	〇	〇	〇	〇	●	●	●	●
Hindlimb															
Ilium	―	―	〇	〇	〇	〇	●	●	●	●	●	●	●	●	●
Ischium	―	―	―	〇	〇	〇	〇	●	●	●	●	●	●	●	●
Pubis	―	―	―	―	〇	〇	●	●	●	●	●	●	●	●	●
Femur	―	〇	〇	〇	●	●	●	●	●	●	●	●	●	●	●
Tibia	―	〇	〇	〇	●	●	●	●	●	●	●	●	●	●	●
Fibula	―	〇	〇	〇	●	●	●	●	●	●	●	●	●	●	●
Patella	―	―	―	―	―	―	―	〇	〇	〇	〇	〇	〇	〇	〇
First metatarsus	―	―	〇	〇	〇	〇	〇	〇	〇	〇	〇	●	●	●	●
Second metatarsus	―	―	〇	〇	●	●	●	●	●	●	●	●	●	●	●
Third metatarsus	―	―	〇	〇	●	●	●	●	●	●	●	●	●	●	●
Fourth metatarsus	―	―	〇	〇	●	●	●	●	●	●	●	●	●	●	●
First phalanx of the first toe	―	―	―	―	〇	〇	〇	●	●	●	●	●	●	●	●
Second phalanx of the first toe	―	―	―	―	〇	〇	〇	〇	〇	〇	〇	●	●	●	●
First phalanx of the second toe	―	―	―	〇	〇	〇	●	●	●	●	●	●	●	●	●
Second phalanx of the second toe	―	―	―	―	〇	〇	〇	●	●	●	●	●	●	●	●
Third phalanx of the second toe	―	―	―	―	〇	〇	〇	〇	〇	〇	〇	●	●	●	●
First phalanx of the third toe	―	―	―	〇	〇	〇	●	●	●	●	●	●	●	●	●
Second phalanx of the third toe	―	―	―	〇	〇	〇	〇	●	●	●	●	●	●	●	●
Third phalanx of the third toe	―	―	―	―	〇	〇	〇	●	●	●	●	●	●	●	●
Fourth phalanx of the third toe	―	―	―	―	〇	〇	〇	〇	〇	〇	〇	●	●	●	●
First phalanx of the fourth toe	―	―	―	〇	〇	〇	〇	●	●	●	●	●	●	●	●
Second phalanx of the fourth toe	―	―	―	〇	〇	〇	〇	〇	●	●	●	●	●	●	●
Third phalanx of the fourth toe	―	―	―	―	〇	〇	〇	〇	●	●	●	●	●	●	●
Fourth phalanx of the fourth toe	―	―	―	―	〇	〇	〇	〇	〇	●	●	●	●	●	●
Fifth phalanx of the fourth toe	―	―	―	―	―	〇	〇	〇	〇	〇	〇	●	●	●	●

―, Stained with neither Alcian Blue 8GX nor Alizarin Red S; 〇, stained blue with Alcian Blue 8GX; ●, partlialy stained red with Alizarin Red S.

a Newly hatched chicks.

### 3.1 Stage I: 3‐day embryos (NNT stage 19; Figure 1a)

Only the vertebral column was stained blue. Blue staining was observed in the vertebral column around the entire notochord except for the coccygeal region.

### 3.2 Stage II: 4‐day embryos (NNT stage 21; Figure 1b)

*Skull*. The basioccipital, exoccipital, and basisphenoid were stained blue.

*Vertebrae*. All the centrums were chondrified as blue staining appeared in the coccygeal region. Bilateral blue staining on each centrum appeared in the bases of the vertebral arches from the cervical to lumbosacral regions.

*Forelimb*. The humerus was stained blue.

*Hindlimb*. The femur, tibia, and fibula were stained blue.

### 3.3 Stage III: 5‐day embryos (NNT stage 23; Figure 1c)

*Skull*. The orbitosphenoid, petrosal, trabeculae cartilage, quadrate, and mandibular cartilage were stained blue. In the hyoid apparatus, the basihyal, urohyal, ceratobranchial, and epibranchial were stained blue. Filmy blue‐stained tissue was present around the eye.

*Vertebrae*. The bases of the arches in the coccygeal vertebrae were bilaterally stained blue.

*Ribs and sternum*. Blue staining appeared in the proximal portion from the first to the seventh vertebral ribs.

*Forelimb*. The scapula, coracoid, radius, ulna, and the second and third metacarpals were stained blue.

*Hindlimb*. The ilium and the first‐to‐fourth metatarsals were stained blue

### 3.4 Stage IV: 6‐day embryos (NNT stage 25; Figure 1d)

*Skull*. The parasphenoid and articular cartilage were stained blue.

*Vertebrae*. All vertebral arches were bow shaped.

*Ribs and sternum*. The first‐to‐seventh vertebral ribs lengthened.

*Forelimb*. Blue staining was present in the first metacarpal. The first phalanges of the second and third digits were stained blue.

*Hindlimb*. Blue‐stained ischium appeared caudally to the ilium. The first phalanx of the second toe and the first and second phalanges of the third and fourth toes were stained blue.

### 3.5 Stage V: 7‐day embryos (NNT stage 27; Figure 1e)

*Skull*. The mesethmoid was stained blue. The quadratojugal, supra‐angular, and angular were stained red in each bone.

*Vertebrae*. Henceforth, the cervical, thoracic, lumbosacral, and coccygeal vertebrae were divided into the upper, medial, and lower regions. The bilateral ribs on the ventral side of the medial‐to‐lower cervical vertebrae were stained blue.

*Ribs and sternum*. A pair of blue‐stained sternal rudiments was observed in the dorsolateral thorax wall. The third‐to‐sixth sternal ribs were stained blue. The vertebral and sternal ribs were not yet connected.

*Forelimb*. The proximal surfaces of the clavicles were stained red but no staining was seen in the ventral midline. All phalanges were visible. The first and second phalanges of the first digit and the second phalanx of the second digit were all stained blue. The central portions of the humerus, radius, ulna, and the second metacarpal all turned red.

Length of the humerus (LH): 1.99 ± 0.20 mm. Percentage of ossified (red) region in the humerus (PH): 22%.

Length of the ulna (LU): 1.73 ± 0.20 mm. Percentage of ossified (red) region in the ulna (PU): 23%.

*Hindlimb*. The first and second phalanges of the first toe, the second and third phalanges of the second toe, and the third and fourth phalanges of the third and fourth toes were stained blue. The central portions of the femur, tibia, fibula, and the second‐to‐fourth metatarsals turned red. Two proximal carpals were stained blue.

Length of the femur (LF): 2.17 ± 0.26 mm. Percentage of ossified (red) region in the femur (PF): 25%.

Length of the tibia (LT): 2.29 ± 0.24 mm. Percentage of ossified (red) region in the tibia (PT): 25%.

### 3.6 Stage VI: 8‐days embryo (NNT stage 29; Figure 1f)

*Skull*. The squamosal, frontal, prefrontal, nasal, premaxillary, maxillary, pterygoid, jugal, and splenial were partially stained red. In the hyoid apparatus, the entoglossal was stained blue and the central portion of the ceratobranchial turned red. The tip of the epibranchial curved superiorly and was level with the exoccipital.

*Vertebrae*. The bilateral cervical ribs were stained blue on the ventral side of the upper region of the cervical vertebrae. The lumbosacral vertebrae were shorter and thicker than the others. The transverse processes in the lower parts of the lumbosacral vertebrae were stained blue.

*Ribs and sternum*. Pairs of sternal rudiments and clavicles fused at the ventral midline. The uncinated processes of the second‐to‐fifth vertebral ribs were stained blue.

*Forelimb*. The scapula and the central portion of the third metacarpal turned red.

LH: 2.68 ± 0.26 mm. PH: 33%.

LU: 2.29 ± 0.18 mm. PU: 41%.

*Hindlimb*. All phalanges were present. The fifth phalanx of the fourth toe was stained blue. Red staining in the fibula spread from the central portion to the distal tip. The cranial ends of the pubis and the ischium fused with the caudal end of the ilium. The two blue‐stained proximal carpals first appearing at 7‐day embryos fused. The distal carpal was stained blue.

LF: 3.13 ± 0.17 mm. PF: 40%.

LT: 3.29 ± 0.30 mm. PT: 43%.

### 3.7 Stage VII: 9‐day embryo (NNT stage 31; Figure 1g)

*Skull*. The peripheries of the basioccipital and exoccipital joints turned red. The parietal dentary and prearticular were stained red. The quadrate turned red. The epibranchial extended superiorly and its tip was level with the petrosal.

*Ribs and sternum*. The central portions of the third‐to‐sixth vertebral ribs turned red.

*Forelimb*. The coracoid partially turned red.

LH: 3.85 ± 0.38 mm. PH: 40%.

LU: 3.38 ± 0.33 mm. PU: 42%.

*Hindlimb*. The ilium and pubis partially turned red. The proximal ends of the second‐to‐fourth metatarsals began to fuse with the distal carpal. A cartilaginous process extended distally from the dorsomedial portion of the proximal metatarsals. A small crescent‐shaped bone located near the distal end of the tibia was stained blue. The phalanges of the toes started to ossify. The central portions of the first phalanges of the second and third toes turned red.

LF: 4.55 ± 0.38 mm. PF: 49%.

LT: 5.20 ± 0.57 mm. PT: 54%.

### 3.8 Stage VIII: 10‐day embryo (NNT stage 32; Figure 1h)

*Skull*. The parietal was stained red. Fourteen sclerotic bones were stained red around the cornea.

*Ribs and sternum*. The first, second, and seventh vertebral ribs partially turned red. The uncinate process of the sixth vertebral rib and manubrium and crest of the sternum were stained blue.

*Forelimb*. The phalanges of the digits started to ossify. The first phalanx of the first digit and the first and second phalanges of the second digit turned red.

LH: 4.38 ± 0.26 mm. PH: 48%.

LU: 3.78 ± 0.21 mm. PU: 51%.

*Hindlimb*. The ischium partially turned red. The patella was stained blue. The nodules of the proximal carpals fused with the distal end of the tibia. A cartilaginous process grew distally from the dorsolateral portion of the proximal metatarsals. The first phalanx of the first toe, the second phalanx of the second toe, the second and third phalanges of the third toe, and the first phalanx of the fourth toe turned red. The caudolateral part of the ilium bent ventrally.

LF: 5.33 ± 0.38 mm. PF: 57%.

LT: 6.53 ± 0.49 mm. PT: 61%.

### 3.9 Stage IX: 11‐day embryo (NNT stage 33; Figure 1i)

*Skull*. The supraoccipital, parasphenoid, basisphenoid, orbitosphenoid, and petrosal partially turned red. The central portion of the basioccipital turned red and the existing staining widened. Red staining of the vomer was observed.

*Vertebrae*. Ossification of the vertebrae began. The centrums of the upper region of the cervical vertebrae and all the thoracic vertebrae partially turned red.

*Ribs and sternum*. All ribs have appeared as the seventh sternal rib was stained blue.

*Forelimb*.

LH: 4.64 ± 0.41 mm. PH: 56%.

LU: 3.93 ± 0.58 mm. PU: 58%.

*Hindlimb*. The second and third phalanges of the fourth toe partially turned red.

LF: 6.09 ± 0.52 mm. PF: 65%.

LT: 7.24 ± 0.60 mm. PT: 67%.

### 3.10 Stage X: 12‐day embryo (NNT stage 34; Figure 1j)

*Skull*. The parasphenoid and mesethmoid fused. The trabecular cartilage became part of the maxilla and the mesethmoid. The frontal extended caudally and joined the parietal at the level of the eye.

*Vertebrae*. The centrums of the medial‐to‐lower regions of the cervical vertebrae and the upper region of the lumbosacral vertebrae partially turned red. The arches of all the cervical vertebrae and the upper region of the thoracic vertebrae also partially turned red. There was partial red staining in the ribs of all the cervical vertebrae.

*Forelimb*.

LH: 5.13 ± 0.20 mm. PH: 63%.

LU: 4.30 ± 0.18 mm. PU: 65%.

*Hindlimb*. The second‐to‐fourth metatarsals were compressed in a row. The fourth phalanx of the fourth toe partially turned red.

LF: 7.23 ± 0.38 mm. PF: 70%.

LT: 8.76 ± 0.50 mm. PT: 73%.

### 3.11 Stage XI: 13‐day embryo (NNT stage 35; Figure 1k)

*Vertebrae*. The centrums of the medial regions of the lumbosacral vertebrae partially turned red. The arches of the medial regions of the thoracic vertebrae also partially turned red.

*Ribs and sternum*. The central portions of the third‐to‐fifth sternal ribs turned red.

*Forelimb*.

LH: 5.37 ± 0.32 mm. PH: 64%.

LU: 4.63 ± 0.21 mm. PU: 63%.

*Hindlimb*.

LF: 8.00 ± 0.53 mm. PF: 72%.

LT: 10.20 ± 0.74 mm. PT: 74%.

### 3.12 Stage XII: 14‐day embryos (NNT stage 36; Figure 1l)

*Skull*. The rostral part of the mesethmoid turned red. In the eye, 14 enlarged sclerotic bones were distributed in an annular pattern, forming the sclerotic ring.

*Vertebrae*. The centrums of the lower region of the lumbosacral vertebrae and the upper region of the coccygeal vertebrae partially turned red. Red staining appeared in the arches of the lower regions of the thoracic vertebrae and the upper regions of the lumbosacral and coccygeal vertebrae. The transverse process of the lower region of the lumbosacral vertebrae partially turned red.

*Ribs and sternum*. The distal ends of the vertebral ribs and the proximal ends of the sternal ribs fused. The sixth sternal rib turned red.

*Forelimb*. The second phalanx of the first digit and the first phalanx of the third digit partially turned red. Ossification of all bones was complete except for the first metacarpal, which did not ossify until hatching.

LH: 5.75 ± 0.26 mm. PH: 70%.

LU: 4.89 ± 0.29 mm. PU: 68%.

*Hindlimb*. The first metatarsal partially turned red. Red staining was present in the second phalanx of the first toe, the third phalanx of the second toe, the fourth phalanx of the third toe, and the fifth phalanx of the fourth toe. All bones showed complete ossification except for the patella, which was not ossified until hatching.

LF: 9.11 ± 0.76 mm. PF: 78%.

LT: 11.80 ± 0.89 mm. PT: 78%.

### 3.13 Stage XIII: 15‐day embryos (NNT stages 37; Figure 1m)

*Skull*. The articular partially turned red. The prefrontal and frontal fused.

*Vertebrae*. The arches of the medial‐to‐lower regions of the lumbosacral vertebrae and the medial region of the coccygeal vertebrae turned red.

*Ribs and sternum*. Red staining appeared in the seventh sternal rib. The central portions of the laterocranial and inner and outer laterocaudal processes also turned red.

*Forelimb*.

LH: 5.97 ± 0.59 mm. PH: 75%.

LU: 5.39 ± 0.39 mm. PU: 73%.

*Hindlimb*. The second‐to‐fourth metatarsals fused, forming the tarsometatarsus.

LF: 10.80 ± 0.96 mm. PF: 83%.

LT: 14.40 ± 1.10 mm. PT: 83%.

### 3.14 Stage XIV: 16‐day embryos (NNT stage 38; Figure 1n)

*Sternum*. The uncinate processes of the second‐to‐sixth ribs partially turned red.

*Forelimb*.

LH: 6.25 ± 0.47 mm. PH: 75%.

LU: 5.39 ± 0.43 mm. PU: 74%.

*Hindlimb*.

LF = 10.80 ± 0.92 mm. PF = 85%.

LT = 14.40 ± 1.10 mm. PT = 86%.

### 3.15 Stage XV: Newly hatched chicks (NNT stage 39; Figure 1o)

*Skull*. The epibranchial lengthened and its tip reached the parietal. The fontanelle remained open between the parietal and the frontal. The basioccipital turned completely red.

*Vertebrae*. The centrums of the medial‐to‐lower regions of the coccygeal vertebrae partially turned red. The arches of the lower regions of the coccygeal vertebrae also partially turned red.

*Ribs and sternum*. Red staining appeared in the sternum body.

*Forelimb*.

LH: 6.48 ± 0.51 mm. PH: 76%.

LU: 5.71 ± 0.35 mm. PU: 78%.

*Hindlimb*.

LF: 11.50 ± 0.61 mm. PF: 87%.

LT: 15.00 ± 0.67 mm. PT: 88%.

## 4 DISCUSSION

### 4.1 General skeletal development of the blue‐breasted quail

The timing and order of chondrification and ossification in almost all bones including the skull, vertebrae, ribs and sternum, and fore and hindlimbs were schematically represented in Tables 1, 2, 3, 4. The developmental features of the above four skeletal systems were summarized as below.

For the skull system, many bones, for example, squamosal, parietal, frontal, prefrontal, nasal, premaxillary, maxillary, palatine, vomer, pterygoid, jugal, quadratojugal, dentary, supra‐angular, angular, splenial, and prearticular, were membranous bones, and they ossified directly between 7 and 11 days of incubation (NNT stages 27–33) as shown in Table 1. All the skull bones had appeared by 11 days of incubation (NNT stage 33). The hyoid apparatus remained cartilaginous except for the ceratobranchial at hatching (NNT stage 39).

Vertebrae system revealed the earliest appearance of cartilage among the four skeletal systems. As shown in Table 2, most bones chondrified between 3 and 5 days of incubation (NNT stages 19–23). In contrast, they ossified at relatively late stages (11–17 days of incubation; NNT stages 33–39). Ossification progressed from the cervical to coccygeal regions. In the lumbosacral and coccygeal vertebrae, ossification began at the upper region. Chondrification and ossification mostly occurred from the centrum to the vertebral arch.

Ribs and sternum showed the latest appearance of cartilage in the four skeletal systems. As shown in Table 3, most bones chondrified between 5 and 8 days of incubation (NNT stages 23–29). Ossification of vertebral and sternal ribs started at the medial region and progressed to the proximal and distal region. Sternum manubrium and crest remained cartilaginous at hatching (NNT stage 39).

Fore and hindlimbs revealed the earliest appearance of ossified regions among the four skeletal systems. As shown in Table 4, most bones started ossification between 7 and 10 days of incubation (NNT stages 27–32). Chondrification and calcification progressed from proximal to distal bones. The first metacarpus and patella appeared at 6 and 9 days of incubation (NNT stages 25 and 31), respectively, and remained cartilaginous at hatching (NNT stage 39).

### 4.2 Comparative ossification sequence of blue‐breasted and Japanese quail

A comparison of the skeletal development between the blue‐breasted quail and the Japanese quail was conducted on the basis of the skeletogenesis list reported in this study. For the purposes of this comparison, the skeletogenous stages of the Japanese quail were consulted (Nakane & Tsudzuki, 1999). The developmental differences and skeletogenous heterochrony between blue‐breasted and Japanese quail embryos are presented below.

Skeletogenous heterochrony between these two species was the most pronounced for the skull system. The development times of the occipital bones were highly variable between the two quail species. The basioccipitals of the blue‐breasted quail embryos chondrified later than those of the Japanese quail embryos but calcified earlier than them. Chondrification and calcification of the exoccipital occurred earlier in the blue‐breasted quail embryos than in the Japanese quail embryos. Chondrification of their supraoccipitals was occurred in the same period but calcification was faster in the blue‐breasted quail than in the Japanese quail. Chondrification and calcification of the parasphenoid and the basisphenoid were delayed, but orbitosphenoid calcification was accelerated in the blue‐breasted quail. The temporal development was almost the same between two quail species but slightly delayed for blue‐breasted quail in the part of petrosal. Calcification of the other membranous and mandibular bones occurred at the same time or earlier than it did in the Japanese quail. The frontal, prefrontal, premaxillary, maxillary, quadrate, supra‐angular, angular, splenial, and prearticular appeared earlier in the blue‐breasted quail than they did in the Japanese quail. The only exception was the dentary. For the hyoid apparatus, the basihyal, urohyal, ceratobranchial, and epibranchial chondrified later in the blue‐breasted quail than they did in the Japanese quail. Calcification of the ceratobranchial was delayed in the blue‐breasted quail embryos.

In the cervical vertebrae of the blue‐breasted quail embryos, calcification started from the centrums of the upper region at 11 days of incubation (NNT stage 33) and was completed within 1 day. However, in the Japanese quail embryos, calcification began from the centrums of the medial region at 10 days of incubation (ASE stage 38; Ainsworth, Stanley, & Evans, 2010) and progressed gradually over 4 days. In the thoracic vertebrae of the blue‐breasted embryos, all centrums calcified simultaneously. On the other hand, for the Japanese quail embryos, calcification occurred in the medial region before the upper and lower regions. Development of the lumbosacral vertebrae was virtually identical in both species. For the coccygeal vertebrae, calcification of the centrums in the upper region and the arches of the upper‐to‐medullary regions occurred earlier in the blue‐breasted quail than the Japanese quail. In contrast, the centrums of the medial‐to‐lower regions calcified later in the blue‐breasted quail embryos than they did in the Japanese quail embryos.

The vertebral ribs chondrified earlier in the blue‐breasted quail embryos than they did in the Japanese quail embryos. However, they calcified almost simultaneously in both quail species. In contrast, the uncinate processes chondrified at almost the same time in both species but calcified later in the blue‐breasted quail than in the Japanese quail. Chondrification and calcification of the sternal ribs were delayed in the blue‐breasted quail embryos. The exception was the third sternal rib, which chondrified earlier in the blue‐breasted quail. Fusion of the sternum body pair occurred at 8 days of incubation in the blue‐breasted quail (NNT stage29) but at 9 days of incubation in the Japanese quail (ASE stage 36). Calcification of the sternum body and the uncinate process of the sixth vertebral rib occurred in newly hatched blue‐breasted quail but not in newly hatched Japanese quail.

The calcification patterns of the terminal phalanges of the second digit in the forelimb and the first‐to‐fourth toes in the hindlimb were significantly different between the two quail species. In the blue‐breasted quail embryos, these phalanges calcified much later than the other phalanges. On the other hand, these phalanges calcified slightly late but did nonetheless occur in Japanese quail embryos. The lag time between the calcification of the first and second (terminal) phalanges of the first toe was 4 days in the blue‐breasted quail but only 1 day in the Japanese quail. Calcification of the first metatarsal was delayed in the blue‐breasted quail embryos. The chondrification times of all fore and hindlimb bones were almost equal for both quail species.

Hogg (1980) reported that the order in which bones ossify during embryogenesis shows a species‐specific pattern in birds. Such sequence variability between species as well as lineages provides mounting evidence that adult morphology influences the order of ossification (Maxwell, 2009; Rippel, 1993; Sánchez‐Villagra, Goswami, Weisbecker, Mock, & Kuratani, 2008). Moreover, comparative ossification sequence data have been further examined for its potential phylogenetic utility (Maisano, 2002; Maxwell & Larsson, 2009; Sánchez‐Villagra, 2002; Schoch, 2006). Therefore, further accumulation of data for ossification sequences of various avian species is expected.

## 5 CONCLUSION

In this study, a list of normal embryonic skeletogenous development of the blue‐breasted quail was established. A comparative study revealed that for all skeletal systems, there was substantial skeletogenous heterochrony between the blue‐breasted quail and the Japanese quail. These embryonic skeletogenous stages of the blue‐breasted quail could serve as a reference standard in the fields of developmental biology and teratological testing as well as in the investigation of mutation with skeletal defects and in the study of skeletogenous mechanisms through manipulation of their genome. Moreover, comparative ossification sequence of the blue‐breasted quail and Japanese quail described here would provide insight into their phylogenetic relations and evolutionary history.
